# Bioaccessibility of Mineral Nutrients in Plain Green Spanish-Style Manzanilla Table Olives Packaged in Nutrient Salt Mixtures

**DOI:** 10.3390/foods13172671

**Published:** 2024-08-24

**Authors:** Antonio López-López, José María Moreno-Baquero, Antonio Garrido-Fernández

**Affiliations:** Food Biotechnology Department, Instituto de la Grasa (IG), CSIC, Campus Universitario Pablo de Olavide, Edificio 46, Ctra. Utrera km 1, 41013 Sevilla, Spain; jose.moreno.baquero@gmail.com (J.M.M.-B.); agarrido@ig.csic.es (A.G.-F.)

**Keywords:** bioaccessibility, green Manzanilla table olive packaging, sodium substitution, calcium, potassium, magnesium

## Abstract

Table olives are high in salt, which can negatively impact cardiovascular health. It is essential to reduce their salt content to mitigate such risk. The objectives of the study were to develop an appropriate protocol to determine mineral bioaccessibility in green Spanish-style Manzanilla table olives and to use it to evaluate, for the first time, the effects of replacing 50% NaCl in the packaging brine with KCl, CaCl_2_, and MgCl_2_ on this characteristic. After testing, Miller’s protocol with a post-digestion re-extraction was chosen. The mineral bioaccessibility found was as follows: Na, 93–98%; K, 94–100%; Ca, 19–27% (the lowest accessibility); Mg, 78–91% (moderately accessible); and non-added P, 55–67%. Bioaccessible amounts (mg/100 g pulp) of added minerals in runs were 151–503 for K, 53–109 for Ca, and 54–143 for Mg. The bioaccessible mineral vs. salt concentrations were modelled and plotted using RSM, illustrating the possible predictions from the tested range of combinations. The bioaccessibility of Ca and Mg was approximately 70% and 15% lower than the values indicated on the label based on the chemical analysis. The results discourage Ca fortification in packaging and suggest including bioaccessibility, markedly influenced by the food matrix, on the label for accurate nutritional information.

## 1. Introduction

In recent decades, table olive consumption has surged due to their appealing appearance and versatile presentation options like pitting, slicing, and stuffing. Global production now reaches about three million tons annually, extending beyond Mediterranean regions [1]. The popularity of table olives is fuelled by their mass production and diverse presentation forms, with whole (plain) Manzanilla green Spanish-style olives being particularly popular for their shape, colour, and taste. Processing these olives involves sodium-based treatments, including lye (2–3% NaOH) and brine (6–12% NaCl), for fermentation/storage [2], along with other ingredients like monosodium glutamate, sorbates, and benzoates, which contribute to their sodium content at packaging [3].

There is a global consensus on the harmful effects of excessive sodium consumption, primarily from processed foods. A recent meta-analysis [4] found that high sodium intakes increases the risk of cardiovascular diseases (CVDs) by 6% per gram of salt consumed. In the USA, health authorities warn that excessive sodium intake can increase blood pressure and the risk of heart disease and stroke, with primary sources including sandwiches, rice, pasta, processed vegetables, meat, poultry, seafood, and pizzas [5]. The recommended daily sodium intake in the USA is 2.3 g Na/day [6]. Similar concerns exist in Europe, where the EU has established a reference daily intake (RDI) of 2.4 g Na/day [7]. The European Commission [8] identified bread and meat products as high in salt, leading to campaigns and legislation to reduce salt in these products. The most affected products are bread (Belgium, Croatia, Netherlands, Portugal, and Slovakia), cheese and processed meat (Bulgaria), cereal products (Finland), tomato juice and concentrates (Greece), and bakery products (Hungary) [8]. Although table olives have low per capita consumption in the EU, their high sodium content, except in ripe olives, undermines their nutritional benefits, such as monounsaturated fatty acids, vitamin E, or polyphenols [9]. Green Spanish-style table olives have sodium levels ranging from 1.44 g/100 g pulp to 1.72 g/100 g pulp [10], contributing 60–72% of the daily salt intake (EU), highlighting the need for reducing salt in table olives.

Previous studies on salt replacement primarily focused on the fermentation and storage phases of processing [11,12]. However, modifying the green Spanish-style processing method is challenging due to the high risk of spoilage [2]. Conversely, since most table olive presentations are pasteurised nowadays [3], salt substitution during packaging is a more favourable alternative. This approach has led to new products with various sensory characteristics [13] that are low in sodium and enriched in potassium, calcium, and magnesium (for simplicity, minerals will henceforth be referred to as by their symbols) [14]. These products have improved nutritional profiles, as their corresponding dietary labels indicate. Sensory evaluations consistently revealed that the best-scoring olives typically contained about 50% less NaCl than standard commercial products [15,16]. The challenge now is to determine how to implement this innovation and study how different combinations of other salts influence bioaccessibility. To address this, the response surface methodology (RSM) is commonly used. The RSM economises on experimental labour while accurately predicting and optimising within the experimental region (see Materials and Methods for a detailed explanation of its use in this work).

Nutrient utilisation by humans depends on nutrient content and the food matrix, interfering dietary compounds, and gut functionalities and characteristics, which can affect nutrient release and absorption. Bioaccessibility estimation, measuring the proportion of minerals available for absorption after digestion, provides insight into this potential. For instance, preliminary tests on ripe table olives showed high bioaccessibility for Fe (45%) and P (60%) but low bioaccessibility for Ca (20%) [17]. Estimating Na bioaccessibility proved challenging due to the high salt abundance, even in ripe olives with lower Na content than other olive presentations. The procedures for assessing bioaccessibility are based on the equilibrium between the supernatant and solid residue, so minerals in high abundance are poorly extracted, requiring additional re-extraction for accurate results. In green Spanish-style olives, the proportion of Na, along with K, Ca, and Mg, when added, is high, necessitating methodological adjustment to improve their solubilisation. This survey tested strategies from other studies addressing intense nutrient exchanges between phases during gastrointestinal passage [18].

This study investigated the effect on mineral bioaccessibility of partially substituting NaCl (at 50%) with KCl, CaCl_2_, and MgCl_2_ in the brines used for packaging green Spanish-style Manzanilla table olives. First, protocols were developed to address the high Na content of the products and evaluate the bioaccessibility of key mineral macronutrients. Next, the various concentrations of these salts in the packaging brine were related by the RSM to the mineral bioaccessibility and their contribution to the reference daily intake (RDI). Finally, these findings were processed to show the relationship between the mineral content listed on product labels and the amounts consumers could actually absorb.

## 2. Materials and Methods

### 2.1. Experimental Design

Green Spanish-style Manzanilla olives, with approximately 240 fruits per kilogram, were prepared by JOLCA SA in Huevar, Sevilla (Spain), and maintained in a 10% NaCl brine. A drum containing 20 kg of these olives was transported to the Instituto de la Grasa (IG), CSIC, Sevilla (Spain), and deposited in a refrigerated room. The olives underwent desalting at 8 ± 1 °C to reduce salt content in moisture to 2.5% (g/100 mL). After desalting, the olives showed a pulp proportion of 84.58% (*w*/*w*) and moisture in the pulp of 67.84% (*w*/*w*). These olives were then packaged in glass containers (170 g olives/130 mL brine), using a brine with 25 g/L NaCl (sodium chloride PA-ACS-ISO, Panreac) and 5 g/L of pure lactic acid, which also contained diverse mixtures of KCl (potassium chloride PA-ACS-ISO, Panreac) (range 5–15 g/L), CaCl_2_ (calcium chloride 2-hydrate powder PA-ACS, Panreac) (0–10 g/L), and MgCl_2_ (magnesium chloride 6-hydrate PRS-CODEX, Panreac) (0–10 g/L) (Table 1), the sum of which was constrained to 25 g/L. The design (a simplex lattice) was generated using Design Expert 13.0 (Stat-Easy Inc. Minneapolis, USA). To reach the expected mineral concentrations, the proportions of the chloride salts were readjusted considering the olive/brine ratio in the containers and the hydration degree of every chloride salt. Overall, the sum of the chloride salts in the brines was 50 g salts/L and 5 g lactic acid/L, or 5% *w*/*v* and 0.5% *w*/*v*, respectively, as usually expressed in the producer’s sector, regardless of the run, following the Trade Standard for Table Olives recommended for this style [3]. The equilibrated brines had a pH of about 4.0 units in the final product. The assay also included a Control package following the habitual industrial protocol for comparison. The containers were preserved by pasteurisation at 85 °C for 8.5 min (${PU}_{62.4{}^{\circ}C}^{5.25}\geq$ 15) and stored at 20 ± 2 °C for two months in the Instituto de la Grasa facilities (Sevilla, Spain). Then, the containers were sampled for the in vitro bioaccessibility analysis.

### 2.2. Physicochemical Analysis of Brines and Fruits

The titratable brine acidity, combined acidity, and pH measurements were conducted following the methods described by Garrido-Fernández et al. [2]. To determine pulp moisture content, a portion of the olive pulp was dried until constant weight. This drying process was carried out on a stainless-steel plate in an electric oven (Selecta, Dry-Big 2002970, Abrera, Barcelona, Spain) set at 106 °C.

### 2.3. In Vitro Digestion of Olives

**Figure 1:**
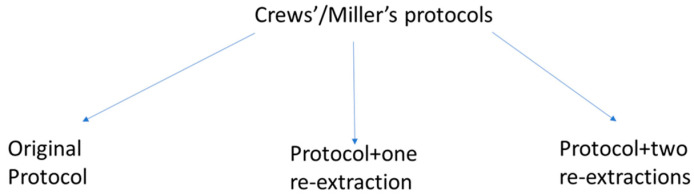
Modifying Crews’ and Miller’s protocols, incorporating one and two wash steps, to adapt the methods to high-Na table olive products.

Previous studies applied various mineralisation methods to ripe table olives, including Miller’s and Crews’ protocols to assess bioaccessibility [17]. These studies concluded that the initial mineralisation method did not significantly affect the results. However, the protocols and the number of extractions notably influenced mineral bioaccessibility, primarily due to the proportion of Na [17]. Given the high levels of Na (especially in the Control) and the fortified K, Ca, and Mg levels in these green Spanish-style olives, it was necessary to modify the existing bioaccessibility protocols to manage them. One logical approach was to increase the number of solubilising solutions. This study compared the original protocols with those using one and two re-extractions of the digested olive pulp (Figure 1).

#### 2.3.1. Miller’s Protocol

This method was based on Miller, Schicker, Rasmussen, and Campen [19] and involved the following sequential steps. Briefly, 2 g of homogenised olive pulp was suspended in 18 mL of water. The pH was adjusted to 2.0 for gastric digestion using 6 N HCl. Then, 625 µL of simulated gastric juice (prepared by dissolving 80 mg of pepsin in 5 mL of 0.1 N HCl) was added to the mixture. The suspension was incubated for 2 h at 37 °C in a shaking water bath incubator set at 110 rpm (WY-200 COD. 5312091, COMECTA, S.A., Abrera, Spain). For the subsequent intestinal digestion, the pH of the gastric digestion mixture was raised to 6.0 with 1 M NaHCO_3_, and 5 mL of simulated intestinal juice (prepared by dissolving 10 mg of pancreatin and 62.5 mg of bile salts in 25 mL of 0.1 M NaHCO_3_) was added. The pH was then adjusted to 7.5 with 1 M NaHCO_3_, and the suspension was incubated at 37° C and 110 rpm for 2 h (WY-200 COD. 5312091, COMECTA, S.A.). After incubation, the digestive enzymes were heat inactivated at 100 °C for 4 min in a stove model (Dry-Big 2002970, J.P. Selecta, Abrera, Spain) and then cooled in an ice bath and centrifuged at 15,550× *g* in a refrigerated (4 °C) instrument (5804R centrifuge, Eppendorf, Hamburg, Germany) for 40 min. The supernatant and solid residue were separated and weighed, and the mineral concentration in each fraction was analysed. The enzymes and bile salts were purchased from Sigma-Aldrich, St. Louis, MI, USA (pepsin from porcine gastric mucosa Cat No. P7000; pancreatin from porcine pancreas Cat No. P1750; α-amylase from porcine pancreas Cat No. A3176; and bile salts Cat No. B8756). A detailed description of this method application can be found elsewhere [17].

#### 2.3.2. Crews’ Protocol

The protocol developed by Crews, Burrell, and McWeeny [20,21] was applied as described below. In summary, 25 g of homogenised olive pulp was weighed and suspended in 50 mL of simulated gastric juice prepared by dissolving 10 mg/mL of pepsin in saline hydrochloric acid (0.15 M NaCl; 0.02 M hydrochloric acid) at pH 1.8. The suspension was incubated at 37 °C and 150 rpm for 2 h (WY-200 COD. 5312091, COMECTA, S.A.). To maintain a pH ≤ 3.5, 6 N HCl was added as necessary. For intestinal digestion, the pH of the suspension was adjusted to 7.4 using a saturated NaHCO_3_ solution, and 50 mL of simulated intestinal juice (prepared by mixing equal volumes of (i) 30 mg/mL pancreatin plus 10 mg/mL of amylase and (ii) 1.5 g/L of bile salts in 0.15 M NaCl) was added. The mixture was again incubated for 2 h at 37 °C and 150 rpm (WY-200 COD. 5312091, COMECTA, S.A.) and centrifuged under refrigeration (4 °C) for 60 min at 30,000× *g* (Sorvall RC6 plus centrifuge, Thermo Scientific, Langenselbold, Germany). The weights and mineral concentrations in the supernatant and the solid residue were calculated, and the mineral bioaccessibility was estimated. As in Miller’s protocol, the enzymes and bile salts used were purchased from Sigma-Aldrich. More details on the application of this method can be found elsewhere [17].

#### 2.3.3. Post-Digestion Re-Extractions

Due to the high NaCl content of green Spanish-style olives, assays for an improved evaluation of the proportion of bioaccessible minerals in table olives were performed. With this objective, the protocols were applied simultaneously to three aliquots of olive pulp, as described in Figure 1. These aliquots were subjected to the standard protocol, standard protocol plus one re-extraction, and standard protocol plus two re-extractions. For the additional re-extractions, the volume of deionised water added was proportional to the usual volumes recommended in each method (10 mL and 125 mL for the Miller’s and Crews’ methods, respectively). In the case of one re-extraction, the solid residue from each standard protocol was combined with the corresponding volume of water, and the resulting suspensions were incubated again for 2 h in a shaking water bath at 37 °C and 110 rpm and then centrifuged for 60 min at 30,000× *g* and 4 °C (Sorvall RC6 plus centrifuge, Thermo Scientific). The supernatants from this step of each protocol were combined with those from their standard applications to form the supernatant of one post-digestion re-extraction. For the assay involving two re-extractions, the process described for one re-extraction was repeated with the digested residues from the first re-extraction.

The mineral contents in the supernatants from (i) the two standard digestions (Miller’s and Crew’s), (ii) supernatants plus one re-extraction, and (iii) supernatants plus two successive re-extractions and their corresponding final solid residues were determined. The mineral bioaccessibilities from these three options (Figure 1) and their contents in the respective solid residues were compared (Table S1).

After comparing the results (as commented in the results and discussion sections), Miller’s technique with one post-digestion extraction was chosen and applied randomly to the packaged olives from the various runs of the experiment. The analyses were performed in triplicate using 100 g of homogenised olive pulp (raw material for the bioaccessibility study) from several glass containers of each run. A blank prepared solely with the reagents was also analysed in parallel with the samples. This blank assessed the mineral contribution of the enzymes and other components used for in vitro digestion.

### 2.4. Wet Mineralisation

Only wet mineralisation was employed in this assay, as previous research [17] showed no significant differences among various mineralisation methods. For the wet mineralisation of supernatants, 20–25 mL of the corresponding solutions were concentrated to 15 mL in a flask. To this concentrate, 5 mL of 65% HNO_3_ was added, and the mixture was heated in a shaking sand bath at 180–220 °C (Combiplac-Sand 6000709; J.P. Selecta, Barcelona, Spain) until the liquid became clear or pale straw-coloured and orange fumes ceased. Subsequently, 5 mL of a mixture of HNO_3_ (65%)/HClO_4_ (60%) in a ratio of 1:4 was added, and the container was heated at 180–220 °C until discolouration and white fumes appeared. The samples were cooled, transferred into a 25 mL volumetric flask, and diluted to volume with distilled deionised water.

For the homogenised olive pulp (raw material) and the digestion solid residues, 2.5 g of the paste was weighed into a 50 mL Erlenmeyer flask, and 5 mL of HNO_3_ (65%) was added. Then, the suspensions underwent the same steps described above for solutions. All reagents were of analytical grade.

### 2.5. Mineral Analysis

In summary, mineral nutrients were quantified in triplicate by atomic absorption spectrophotometry with an air-acetylene flame using a GBC model 932 AA (GBC, Braeside, VIC, Australia) spectrometer equipped with three hollow multi-element cathode lamps: Na and K (Photron, Narre Warren, Victoria, Australia); Cu, Fe, and Mn (GBC, Braeside, VIC, Australia); and Ca, Mg, Cu, and Zn (Photron, Narre Warren, VIC, Australia), following methods described by López López et al. [10,17] and adhering to the analytical conditions recommended by the equipment manufacturer [22]. To mitigate interference and ensure proper ionisation of the air-acetylene flame, lanthanum (0.5%, *w*/*v*) was added to the samples and the calibration standards when analysing Ca and Mg, while K (0.1%, *w*/*v*) and Na (0.1%, *w*/*v*) was added for Na and K, respectively.

Phosphorus was determined using the official AOAC No. 970–39 method for phosphorus in fruits and fruit products, employing the spectrophotometric molybdovanadate method [23]. A Cary UV/visible spectrophotometer model 1E (Varian Australia, Mulgrave, Victoria) was used to measure the absorbance at 400 nm of the yellow phospho-molybdovanadate complex formed in the presence of V^5+^ and Mo^6+^. Each triplicate measurement represented the average of three determinations.

Daily calibration curves were generated from successive dilutions of stock solutions, with interpolation performed after subtracting the blank signal from the samples. Standard solution samples were periodically included in the analysis to ensure method accuracy and reliability.

All glassware used to determine the minerals was immersed in 10% (*w*/*w*) nitric acid overnight and then rinsed several times with distilled deionised water.

### 2.6. Mineral Recovery and Bioaccessibility Estimation

Bioaccessibility and recovery (as a percentage) were estimated using the formulas established by López-López et al. [17]:

(1) $$Bioaccessibility\left(\%\right)=\left(\frac{\left(S-B\right)}{RM}\right)\cdot 100$$

(2) $$Recovery\left(\%\right)=\left(\frac{\left(S-B+SR\right)}{RM}\right)\cdot 100$$

Here, the symbols stand for the concentrations of mineral nutrients in the following substrates: RM, raw material; S, supernatant solutions (including those from the modified protocols); SR, solid residue; and B, blank.

Based on the olives’ mineral contents and the bioaccessibilities determined for each mineral, the contribution (as percentages) of 100 g pulp of plain green Spanish-style Manzanilla olives to the reference daily intake (RDI) of Na, K, Ca, Mg, and P before and after digestion was calculated.

### 2.7. Mineral Nutrient Bioaccessibility, Recovery, and RDI Contribution According to Chloride Salt Mixtures

The effects of the KCl, CaCl_2_, and MgCl_2_ mixtures in packaging brines on the bioaccessibility and contribution to RDI (%) of each mineral were analysed using the response surface methodology (RSM). The procedure involved several steps where significant terms up to the cubic level were retained using the sequential model sum of squares (Type II). For detailed information, readers can consult Myers and Montgomery [24]. The general form of the model, expressed in the canonical (Sheffe’) form, took the following structure:

$$R=\sum_{i=1}^{n}\beta_{i}x_{i}+\sum_{1\leq i<j}^{n}\beta_{ij}x_{i}x_{j}+\sum_{1\leq i<j<k}^{n}C_{ij}x_{i}x_{j}x_{k+\varepsilon }$$

where n represents the number of variables (3 in this case); x_i-j_ denotes the concentrations in the equilibrium of CaCl_2_, KCl, and MgCl_2_ in the packaging brines; R stands for the responses to be modelled, bioaccessibility or RDI (%); β and C_ij_ values are the coefficients to be estimated; and ε is the error term. When no further term significantly increased the variance explained, the model with the retained coefficients (*p*-values for entering and removing variables of *p* ≤ 0.05 and *p* ≤ 0.10, respectively) was then suggested. Its fit was analysed by ANOVA (sum of squares, type III). Models were considered significant when *p*-values were ≤ 0.05. Additionally, parameters such as adjusted R-squared, precision, and standard errors of coefficients were also considered. Finally, equations were formulated in terms of actual coefficients and visually represented through plotting in the simplex.

### 2.8. Statistical Analysis

The impact of various factors, including digestion protocols and their modifications, on mineral bioaccessibility and its contribution to the RDI was studied using the general linear model (GLM), Statistic v. 8.0 [25]. The results were deemed significant at *p* ≤ 0.05 when their mean confidence limits (CLs) did not overlap. The responses to the experimental design were analysed with Design Expert v 13 (Stat-Easy Inc., Minneapolis, MN, USA), as commented in the previous section.

## 3. Results

### 3.1. Assays for Adapting Mineral Bioaccessibility Protocols to Green Table Olives

For protocol adaptation, fermented plain green Spanish-style Manzanilla olives, stored in a 10% NaCl brine, were chosen due to their typically high salt content. The tests focused on the usually occurring macronutrients Na and P, along with K, Ca, and Mg, added during the packaging assay, as these were the only elements present in the supernatants in sufficient concentrations for quantitative analysis. The results of applying Crews’ and Miller’s protocol options, depicted in Figure 1, are reported in Table S1 (average concentration values in the digestion fractions, bioaccessibility, and balances) and Table S2 (average weights of the digestion fractions).

In general, the mineral concentrations in the supernatants decreased as the number of re-extractions increased, resulting in a smaller fraction of the initial minerals remaining in the solid residue. However, the amount of solubilised minerals in the resulting combined supernatants increased with more extensive re-extractions (Table S1). For Na and K, equilibrium between the supernatants and the aqueous phase of the residues could be achieved, as the decrease in mineral content with re-extractions was proportional in both the supernatants and solid residues. However, for Ca, the high content remaining in the solid residues suggests that a sensible portion of Ca could still be bound to pulp components after digestion and numerous re-extractions. The retention of Mg, another divalent mineral, was slightly less pronounced than Ca (Table S1). Considering all fractions, the balance of material showed that the overall mineral recovery ranged from 94.73% to 104.5%, indicating adequate experiment performance. Therefore, the comparisons between standard and modified protocols are reliable.

**Figure 2:**
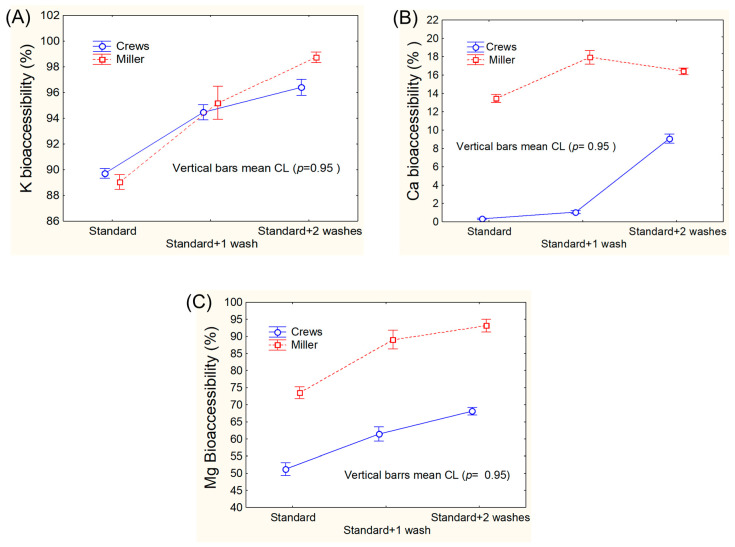
Comparison of the bioaccessibilities of (**A**) potassium, (**B**) calcium, and (**C**) magnesium, according to the standard and modified Crews’ and Miller’s protocols (one and two washes).

**Figure 3:**
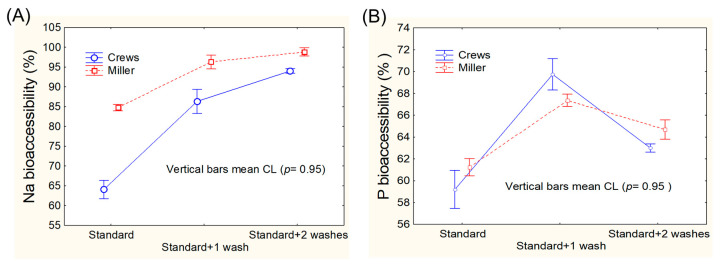
Comparison of the bioaccessibilities of (**A**) sodium, and (**B**) phosphorous, according to the standard and modified Crews’ and Miller’s protocols (one and two washes).

The efficacy of the three modifications was statistically evaluated by analysing the results using a general linear model (GLM), graphically presented in Figure 2 (K, Ca, and Mg) and Figure 3 (Na and P). 

Miller’s protocol led to significantly higher bioaccessibility values for K (only for two re-extractions), Na, Ca, and Mg. For phosphorous, Miller’s protocol led to slightly lower values for one re-extraction but yielded a significantly higher bioaccessibility with two re-extractions (Figure 2 and Figure 3), although the bioaccessibility with two extractions was always lower than that with one re-extraction. Therefore, the standard Miller’s protocol plus one re-extraction strikes a good balance between mineral recovery and protocol complexity without compromising the principles of in vitro digestion.

The adapted protocol was then applied to study mineral bioaccessibility in all runs (including the Control) of the salt substitution assay in green Spanish-style table olive packaging. Given the specific characteristics of this preparation and the potential to apply a similar protocol to ripe olives, this adaptation could be beneficial for studying mineral bioaccessibility in table olives, regardless of style or presentation forms.

### 3.2. Mineral Content in the Pulp of Green Spanish-Style Table Olives Packaged in Chloride Salt Mixtures

The first step in investigating nutrient bioaccessibility is determining nutrient concentrations in the product. The average contents (and standard error) of Na, K, Ca, Mg and P in the olive pulp showed marked differences among the packaging conditions (runs) (Table 2), which will be briefly discussed below.

Regardless of the run, substituting 50% of the NaCl with mixtures of other salts effectively reduced the Na content by about 54% without lowering shelf life when the product was preserved through pasteurisation. However, if pasteurisation is not used, the product’s shelf life could be shortened due to the lower inhibitory power of K, Ca, and Mg than of Na [3]. Additionally, incorporating chloride salts during packaging increased the levels of their minerals in olive pulp (Table 2) and improved their overall health benefits. Specifically, the Na in the olive pulp of final products was reduced from about 14 g/kg pulp in the Control to 7 g/kg pulp in the packages subjected to treatment. Simultaneously, the concentrations of K increased from 100 mg/kg pulp in the Control to a range of 1571–5085 mg/kg pulp in the experimental runs. The concentrations of Ca changed from 603 mg/kg pulp in the Control to 157–4664 mg/kg pulp in the experimental runs. The Mg content varied from 50 mg/kg pulp in the Control to 39–1578 mg/kg pulp in the designed packages. However, the changes in P were negligible, with the concentration changing from 68 mg/kg in the Control to 59–65.38 mg/kg in the runs (Table 2). Readers are referred to previous studies, which included macro- and micronutrients, for a detailed analysis of the effect of adding these chloride salts during the packaging of green Manzanilla Spanish-style table olives on the mineral content in pulp [13].

### 3.3. Bioaccessibility of Mineral Nutrients in Green Spanish-Style Table Olives Packaged with Chloride Salt Mixtures

#### 3.3.1. Mineral Concentrations in Different Fractions after In Vitro Digestion

The packaging conditions used in a study can impact the bioaccessibility of mineral nutrients. To evaluate this, olive samples from each run listed in Table 1 were subjected to Miller’s protocol with an additional post-digestion re-extraction performed in triplicate. Mineral concentrations in the raw material, supernatant, solid residue, and blank, along with their respective fraction weights, were determined for each mineral and are summarised in Table 3 (means and standard errors in parentheses). These values are essential for calculating the mass balance and verifying proper mineral recovery. The sum of the amounts distributed in these fractions should match the mineral content in the raw material, considering the weights provided on the right. However, only the content in the supernatant represents bioaccessibility.

The concentrations of mineral nutrients in the supernatants and solid residues varied among runs, as depicted in Table 2. Following digestion, Ca concentrations in the supernatants were relatively low compared to those in the solid residue, owing to the well-known affinity of Ca for olive pulp. A similar, albeit less pronounced, trend was also observed for Mg, which exhibited sensible retention in the solid residue when introduced to the packaging brine. In contrast, K showed a different pattern, with the majority being recovered in the supernatants and only a tiny amount continuing in the solid residue. The remaining K was primarily associated with the equilibrium between the supernatant and the moisture in the solid residue (Table 2). As for P, a relevant proportion of this element remained in the solid post-digestion residue, consistent with its strong binding to olive pulp components.

#### 3.3.2. Mineral Nutrient Bioaccessibility after In Vitro Digestion

The solubilisation of Na, K, Ca, and Mg can be estimated using the data in Table 2 by considering concentrations, fraction weights, and blank contributions. The bioaccessibility for each mineral (as a percentage) is then calculated by comparing the solubilised amount to the initial content in the raw material. Similarly, the proportion remaining in the solid residue can be estimated. The sum of these bioaccessible and residue proportions should total 100% of the initial mineral content in the olive pulp, confirming the protocol’s accuracy.

Overall, the mineral recoveries (sum of supernatant and solid residue) were close to 100%, indicating the effectiveness of the protocol and its implementation (Table 3). The bioaccessibility of Na (92.97–97.87%) and K (94.47–99.83%) was notably high, suggesting that their presence in the solid residue reflects an equilibrium with the re-extraction supernatants. Therefore, the bioaccessibility of these elements is considered complete. In contrast, Ca showed the lowest bioaccessibility, ranging from 19.41% to 26.97%, with most remaining in the solid residue after digestion.

The bioaccessibility of Mg ranged from 78.38 to 90.77%, indicating a relatively high absorption potential but with somewhat high variability between runs, influenced by packaging conditions other than Na and K. Magnesium was present in solid residues, likely adhering to olive pulp components, but to a lesser extent than Ca.

Phosphorous exhibited a bioaccessibility between 55.44 and 61.78%, indicating significant potential for human absorption, despite not being intentionally added, due to its moderate association with the olive pulp components.

#### 3.3.3. Bioaccessibility and Nutritional Labelling of Minerals

The mineral concentrations in the supernatants, along with their weights and the weight of the digested olive pulp, were used to estimate the bioaccessible mineral content per 100 g of olive pulp and its contribution to the RDI (%) for each run (Table 4) based on the EU recommendations [7]. All runs showed a drastic reduction in Na (around 50%) compared to that in the Control. In contrast, the K content in the Control (10 mg/100 g pulp) was much lower than that in the fortified runs (151–503 mg/100 g pulp), underscoring the effectiveness of the substitution process to in enhancing K content in the olives (8–25% RDI) compared to that in the conventional packaging practices (<1%).

In treatments without CaCl_2_, the Ca content was low (<16 mg/100 g pulp (2% RDI)). However, adding CaCl_2_ markedly increased levels, ranging from 53 mg/100 g pulp (7% RDI) to 109 mg/100 g pulp (14% RDI). The bioaccessibility of Mg in the Control was only 5 mg/100 g pulp (0.01% RDI), but the addition of MgCl_2_ raised it to between 54 mg/100 g pulp (14% RDI) and 143 mg/100 g pulp (38% RDI), exceeding the bioaccessibility of Ca. The bioaccessible P content remained below 4 mg/100 g pulp, with a minimal RDI contribution (<1% RDI) due to its low content and moderate bioaccessibility. These findings highlight the substantial impact of CaCl_2_ and MgCl_2_ fortification on enhancing the mineral content and bioaccessibility of experimental olive products compared to those in conventional packaging (Control).

For obtaining the overall picture (response surface, RS), the bioaccessibilities of K, Ca, and Mg (in mg/100 g olive pulp) were related to the initial proportions of their respective salts in the packaging brines. In this design, cubic terms were aliased and discarded. The functions linking the salt mixture to the bioaccessible mineral amounts and their contributions to the RDI (%) were linearly dependent, implying that their fit parameters were identical. The equations for the different minerals and their contributions to RDI were as follows:

Potassium:

- Model parameters: significant, *p* < 0.0001; lack of fit, *p* = 0.04508; precision, 53
- Amount bioac. (mg/100 g pulp)$=+33.03\cdot \left[KCl\right]+0.24\cdot \left[{CaCl}_{2}\right]-0.31\cdot [{MgCl}_{2}]$
- Contribution to RDI (%) = $+1.651\cdot \left[KCl\right]+0.012\cdot \left[{CaCl}_{2}\right]-0.0155\cdot \left[{MgCl}_{2}\right]$

Calcium:

- Model parameters: significant, *p* < 0.0001; lack of fit, *p* = 0.4508; precision, 53
- Amount bioac. (mg/100 g pulp) $=+0.72\cdot \left[KCl\right]+9.48\cdot \left[{CaCl}_{2}\right]+0.60\cdot [{MgCl}_{2}]$
- Contribution to RDI (%) = $+0.0895\cdot \left[KCl\right]+1.185\cdot \left[{CaCl}_{2}\right]+0.0744\cdot \left[{MgCl}_{2}\right]$

Magnesium:

- Model parameters: significant, *p* < 0.0001; lack of fit, *p* = 0.2158; precision, 95.16
- Amount bioac. (mg/100 g pulp) = $-1.68\cdot \left[KCl\right]-5.27\cdot \left[{CaCl}_{2}\right]+14.88\cdot \left[{MgCl}_{2}\right]+0.55\cdot \left[KCl\right]\cdot \left[{CaCl}_{2}\right]+0.21\cdot \left[{CaCl}_{2}\right]\cdot \left[{MgCl}_{2}\right]$
- Contribution to RDI (%) =$-0.447\cdot \left[KCl\right]-1.406\cdot \left[{CaCl}_{2}\right]+3.968\cdot \left[{MgCl}_{2}\right]+1.470\cdot \left[KCl\right]\cdot \left[{CaCl}_{2}\right]+0.500\cdot \left[{CaCl}_{2}\right]\cdot \left[{MgCl}_{2}\right]$

Despite the model for Mg showing a value close to the limit but a significant lack of fit, it was deemed reasonable due to its high significance (*p* < 0.0001) and precision (53, indicating a high rate of signal to noise). Moreover, it can also be interesting from a technological viewpoint.

**Figure 4:**
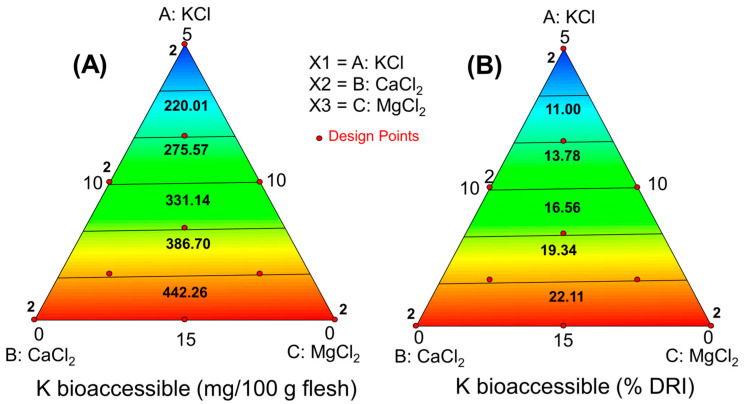
Study of the potassium bioaccessibility of green Spanish-style Manzanilla olives with K, Ca, and Mg chloride mixtures substituting 50% salt during packaging. Predictions as functions of the KCl, CaCl_2_, and MgCl_2_ in the packaging brines, (**A**) bioaccessible amount, and (**B**) contribution (%) to the reference daily intake (RDI).

**Figure 5:**
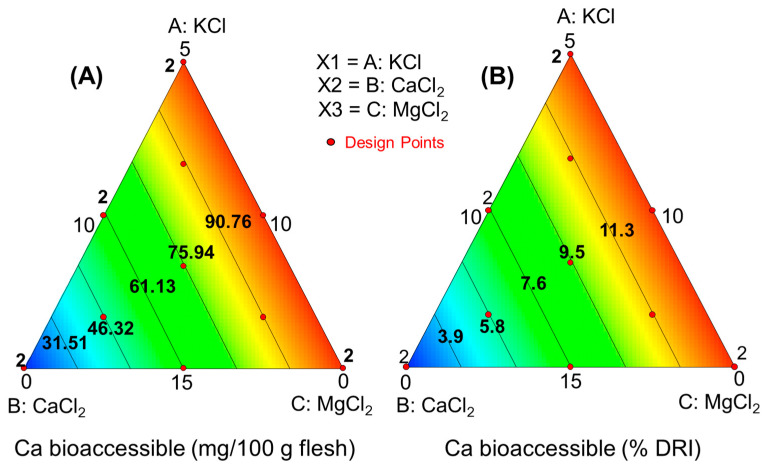
Study of the calcium bioaccessibility of green Spanish-style Manzanilla olives with K, Ca, and Mg chloride mixtures substituting 50% salt during packaging. Predictions as functions of the KCl, CaCl_2_, and MgCl_2_ in the packaging brines, (**A**) bioaccessible amount, and (**B**) contribution (%) to the reference daily intake (RDI).

**Figure 6:**
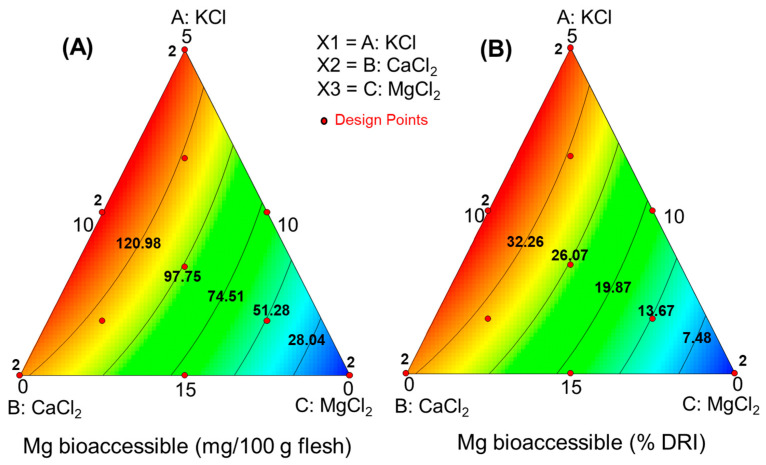
Study of the magnesium bioaccessibility of green Spanish-style Manzanilla olives with K, Ca, and Mg chloride mixtures substituting 50% salt during packaging. Predictions as functions of the KCl, CaCl_2_, and MgCl_2_ in the packaging brines, (**A**) bioaccessible amount, and (**B**) contribution (%) to the reference daily intake (RDI).

These equations were visualised in the simplex (Figure 4, Figure 5 and Figure 6), illustrating how added salts in the brine affect the characteristics of plain green Spanish-style Manzanilla olives and their fortification with K, Ca, and Mg nutrients. The bioaccessible concentrations of K and Ca increased linearly with their respective salt concentrations in the packaging brine. Specifically, bioaccessible concentrations of K can contribute up to 25% (Figure 4B). However, olives fortified with CaCl_2_ were less efficient carriers, as the bioaccessibility of Ca, even at the maximum concentrations, typically reached only 100 mg Ca/100 g pulp (about 12% RDI) (Figure 5A,B).

Fortification with MgCl_2_ shows greater promise, as the bioaccessible content from 100 g pulp can reach up to 130 mg, contributing around 35% of the RDI (Figure 5A,B). Thus, fortifying table olives with K and Mg could make them a notable source of these minerals. However, the low bioaccessibility of Ca makes its fortification less beneficial.

This study highlights that nutrition labels offer only a rough guide for consumers. Nutrient utilisation depends heavily on bioaccessibility, which can vary widely among different foods.

#### 3.3.4. Comparison between Usual Mineral Nutrition Labelling and that Based on Bioaccessibility

Discrepancies between the nutritional mineral information in the plain green Spanish-style Manzanilla table olive label and the bioaccessible mineral macronutrients were assessed by comparing the nutrient quantities in the raw material with their respective bioaccessibilities (Table 4 and Table 5). To enhance clarity, data are presented in two formats: one showing mineral concentrations from direct analysis (expressed as mg/100 g pulp) and the other reflecting bioaccessible minerals (bioaccessible mg of mineral/100 g pulp). For simplicity, units have been omitted from the table.

The data are discussed by comparing the label values, typically based on analytical content, with the lowest and highest bioaccessible concentrations observed in the experiment. This comparison illustrates the range of values encompassed by the study design.

For 100 g pulp, the P content in this study was below the typical nutritional labelling interest, but P in olives is efficiently utilised. In the Control (typical conditions), the P content in pulp was 6.835 mg/100 g by analysis, with a bioaccessible amount of 4.16 mg/100 g pulp (Table 5), indicating 60.86% potential utilisation. Across experimental runs, the average analysed P content was 6.247 mg/100 g pulp, with an average bioaccessible amount of 3.675 mg/100 g, suggesting a 58.82% absorption rate.

For sodium, the values based on direct analysis vs. bioaccessibility in the Control were 1359 mg Na/100 g pulp (typical label) vs. 1329 mg Na bioaccessible/100 g pulp (56.63% vs. 55.38% RDI), showing a close match between both labelling approaches. In the experimental runs, the average Na concentrations from analysis vs. bioaccessibility were 630 mg Na/100 g pulp vs. 586 mg/100 g pulp (26.25–25.50% RDI, respectively) for the lowest content and 662 mg Na/100 g pulp vs. 641 mg/100 g pulp (27.60–27.90% RDI, respectively) for the highest content. These minimal differences are due to the low Na retention in the solid phase after equilibrium following post-digestion re-extraction.

Potassium showed a similar pattern to Na, although with broader ranges due to the inclusion of KCl in the salt mixtures. In the Control, nearly complete utilisation was observed, at 9.95 mg K/100 g pulp compared to 9.80 mg K bioaccessible/100 g pulp (<1% RDI), indicating minimal nutritional interest. In the experimental runs, depending on the KCl concentrations in the packaging brine, the values ranged from 157.16 mg K/100 g pulp and 151.10 mg K bioaccessible/100 g pulp (7.85–7.55% RDI, respectively) for the lowest level to 515.20 mg K/100 g pulp and 502.7 mg K bioaccessible/100 g pulp (25.75–25.14% RDI, respectively) for the highest level (see Table 4 for details).

For Na and K, the differences between the label values and bioaccessible amounts were minimal across all packaging conditions, indicating an almost complete potential utilisation of these minerals by humans. In contrast, the situation for Ca was quite different. In the Control, the utilisation was low: 60.86 mg Ca/100 g pulp by analysis vs. 16.1 mg Ca bioaccessible/100 g pulp (contributions of 7.61% vs. 2.02% RDI, respectively). In the experimental runs, depending on the CaCl_2_ concentrations in the packaging brine, values ranged from 57.751 mg Ca/100 g pulp vs. 14.70 mg Ca bioaccessible/100 g pulp (7.22% vs. 1.84% RDI, respectively) for the lowest level to 472.94 mg Ca/100 g pulp vs. 109.20 mg Ca bioaccessible/100 g pulp (59.12% vs. 13.65% RDI, respectively) for the highest level.

The values based on the pulp analyses were markedly higher than the bioaccessible amounts (see Table 4). The difference in Ca that consumers might not absorb ranged from 43.05 (57.751 minus 14.7) mg Ca/100 g pulp to 363.74 (472.936 minus 13.65) mg Ca/100 g pulp, reflecting a reduction in nutritional expectation from 74.54% to 76.91%. Thus, relying on traditional nutrition information could lead to misleading calculations and potentially inadequate Ca intake.

For Mg, the situation is less critical than for Ca but differs from that for Na and K, with bioaccessibility falling between these extremes. In the Control, the differences were minimal: 4.95 mg Mg/100 g pulp vs. 4.00 mg Mg bioaccessible/100 g pulp (1.32% vs. 1.07% RDI, respectively). In the experimental runs, depending on the MgCl_2_ concentration in the packaging brine, values ranged from 5.489 mg Mg/100 g pulp vs. 4.40 mg Mg bioaccessible/100 g pulp (1.46–1.17% RDI, respectively) at the lowest level to 163.796 mg Mg/100 g pulp vs. 141.70 mg Mg bioaccessible/100 g pulp (43.68% vs. 37.78% RDI, respectively) at the highest level.

The non-released Mg amount could range from 0.81 mg/100 g pulp (3.912 minus 3.10) to 22.10 (163.80 minus 141.70) mg/100 g pulp, or 20.71% to 13.49% of the total. This suggests that label information for Mg might mislead consumers, as the actual utilisation, based on bioaccessibility, is lower.

This study highlights the need for better nutritional labelling: in addition to nutrient concentrations, labels should include bioaccessibility data, especially for nutrients with low bioaccessibility.

## 4. Discussion

This study is the first to investigate bioaccessibility in whole (plain) green Spanish-style Manzanilla olives. Given the lack of prior data, the protocol for managing high Na content [10,17] was re-evaluated. It was found that at least one re-extraction was necessary to achieve reasonable solubilisation, with a second re-extraction showing a minimal additional yield and increased labour. Therefore, Miller’s protocol was supplemented with an additional re-extraction using distilled deionised water. Surprisingly, despite the significant Na content difference, this protocol was consistent with the one used for ripe olives [17].

Sodium is essential for cellular homeostasis and physiological function, but excessive dietary Na has been associated with elevated blood pressure and harmful effects on target organs such as the blood vessels, heart, kidneys, and brain [26,27]. Na bioaccessibility in olives was found to be nearly complete (93–98%) (Table 4), and in typical packaging (based on 100 g pulp), it could contribute up to 58% of the RDI. Reducing NaCl in olive packaging by half, thereby lowering its contributing to about 27% of the RDI, could significantly improve consumer health and reduce associated risks and healthcare costs [28]. Ripe olives are distinguished by their high Fe content, which is moderately bioaccessible and makes them a good source of this nutrient element [17]. However, Ca bioaccessibility in ripe olives is low [17]. This study (Table 3) confirms the relatively low calcium bioaccessibility (19–27%) in green Manzanilla Spanish-style olives and potentially in table olives overall. This low bioaccessibility may limit the effectiveness of Ca supplementation in these products, constraining their intended nutritional benefits.

Despite the acidic environment that accelerates olive softening [29], Ca remains tightly bound to organic compounds in the olive pulp, as noted by Jiménez Araujo et al. [30]. Additionally, Ca is considered essential for preserving ripe olives [31], regardless of whether lactate or chloride salts are used. [32]. However, the specific olive compounds to which Ca is linked are poorly understood. While some initial Ca may originate from cytosolic-Ca^2+^ [33], it is unlikely that the added Ca enters the cell cytoplasm of processed fruits. Instead, it likely reacts with cell wall components, primarily pectic fractions (primarily) and hemicelluloses, which are abundant in olive pulp [30]. This hypothesis is supported by findings indicating that the pectic fraction of olive pulp can form stable gels in the presence of Ca [34] and by X-ray diffraction studies by Ferreira et al. [35], who identified the existence of crystalline phases related to Ca–pectic polysaccharide complexes (CPPC) within an amorphous carbohydrate network in olive cell walls. The relative crystallinity is linearly related to the Ca^2+^/galacturonic acid ratio. Therefore, the low bioaccessibility of Ca in olives may be due to the poorly accessible cytosolic Ca^2+^ (within the cells) and its strong binding to pectic substances in the cell walls. 

The human health implications of Ca are not yet fully elucidated. A recent review found no conclusive link between Ca intake and cancer, bone health, or cardiovascular outcomes. However, Ca intake was associated with a slight 2–4 mmHg decrease in systolic blood pressure in pregnant and hypertensive subjects [36]. Conversely, excessive Ca intake (overdoses) may increase the risk of kidney stones, myocardial infarction, and stroke [37].

Research indicates that the P bioaccessibility in Spanish-style olives ranges from 55% to 62%, compared to approximately 60% in ripe olives [17]. This difference may be due to the more intense alkaline treatment used in processing. Phosphorous bioaccessibility is a key area of study because of its essential role in dietary formulations. About 80% of the P in the human body is found in the skeleton, while the remaining 20% is involved in nucleotides, phospholipids, and other phosphorylated compounds crucial for metabolism [38]. The relatively high bioaccessibility of P in olives positions them as a valuable source of this nutrient, especially when compared to the low bioaccessibility of plant-derived phosphorus in whole grains, legumes, peas, nuts, and seeds, where it is often bound to phytates [39].

The bioaccessibility of K in green olives was notably high, ranging from 94.47% to 99.83%. These values are comparable to those found in “high bioaccessible diets” but higher than the 77% reported for a diet rich in fruits and vegetables, which is influenced by differences in the cellular structure of plant foods [40]. The presence of K in olives may counteract the effect of Na, as epidemiological studies and clinical trials have consistently shown an inverse association between K intake and blood pressure [40,41]. Additionally, supplementing K in table olives could help address the K deficit recently identified in the EU population by the EFSA Panel on Nutrition, Novel Foods, and Food Allergens [42].

Compelling evidence suggests that Mg supplementation can reduce the risk of hospitalisation in pregnant women, alleviate the intensity and frequency of migraines, and lower the risk of type 2 diabetes and stroke, according to observational studies [43]. Therefore, fortifying table olives with Mg could offer significant health benefits. In this study, Mg bioaccessibility ranged from 78.38% to 90.77% (Table 4), validating the fortified product as a substantial source of this mineral. Magnesium is moderately present in fruits and vegetables and is primarily absorbed as an ion rather than in complexes [44]. In olive leaves, all tissues, except the bundle sheath and the main vascular bundle, contribute to the bulk of the Mg concentration [45]. Mg plays a critical role in plant cells and cell walls, acting as a catalyst in enzymatic reactions, stabilising cell membranes, and contributing to ion homeostasis. It interacts with ion channels, modulating their activity and controlling the movements of ions such as K^+^, Ca^2+^, and other cations across membranes [46]. The non-bioaccessible portion of Mg in olives may be associated with these enzymes and compounds initially containing Mg, while added Mg may simply dissolve in the pulp moisture or form weak pectic complexes. Additionally, inhibitory components for Mg absorption, such as oxalate, phytic acid, cellulose, and lignin [44], are either absent or present in low proportions in table olives [2].

The investigation into the bioaccessibility of K, Ca, and Mg across different salt mixture compositions demonstrated that K and Mg can substantially contribute to dietary intake, achieving more than 15% of the RDI, which supports nutritional claims. However, incorporating Ca was less efficient due to its low bioaccessibility, consistently contributing less than 15% of the RDI in the proportions used in this study, thereby preventing any nutritional or health claims regarding Ca. Nevertheless, using Ca in table olives may offer technological benefits [2]. Given its low bioaccessibility, including information about the actual Ca content and bioaccessibility on the nutritional labels could be beneficial, as the current labelling may mislead consumers in their dietary choices.

## 5. Conclusions

For the first time, this study investigated the bioaccessibility of mineral nutrients in green Spanish-style Manzanilla table olives. The research focused on developing an appropriate protocol and examining the impact of substituting 50% NaCl with KCl, CaCl_2_, and MgCl_2_ salt mixtures during packaging on mineral bioaccessibility. For assessing bioaccessibility, Miller’s protocol with a post-digestion re-extraction was selected as a balanced approach between mineral recovery and technical complexity.

The results showed that the bioaccessibility of Na (93–98%) and K (94–100%) from green Spanish-style Manzanilla olives was notably high, indicating that humans may almost entirely absorb these elements. In contrast, Ca exhibited the lowest bioaccessibility, ranging from 19% to 27%, likely due to its strong binding with olive pulp components. Magnesium had moderate bioaccessibility, ranging from 78% to 91%, with less fixation to olive pulp than Ca. Non-added P was also relatively bioaccessible (55–67%), suggesting that olives could be a potential P source for humans.

The bioaccessible amounts of added minerals were related to the concentrations of salts in the packaging brine by the RSM, with models plotted for easier understanding and application. All experimental runs achieved about a 50% salt reduction while markedly increasing K from 10 mg/100 g pulp in the Control to 151–503 mg/100 g pulp across the different runs, Ca from less than 16 mg/100 g pulp to 53–109 mg/100 g pulp, and Mg from 5 mg/100 g pulp to 54–143 mg/100 g pulp. However, due to the poor bioaccessibility of Ca (mainly) and Mg, their potentially usable amounts in green Spanish-style olives for humans were about 70% and 15% lower, respectively. These findings open a new research avenue for reducing salt content in table olives without compromising current processing technology, which is crucial for preventing spoilage during fermentation. Additionally, the study suggests enhancing the health benefits of table olives by reducing salt content while increasing K, Ca, and Mg levels. Applying similar innovations to other commercial presentations could naturally extend these benefits across the sector, potentially improving the image of table olives as a less salty product and broadening their appeal.

The results discourage Ca fortification unless required for other technological reasons. They also highlight the importance of including bioaccessibility information on nutritional labels in addition to contents to provide accurate nutritional information, particularly for nutrients with low bioaccessibility.

## Figures and Tables

**Table 1:** Simplex lattice design used to study the mineral bioaccessibility of green Spanish-style Manzanilla olives with K, Ca, and Mg chloride mixtures substituting 50% salt during packaging. Actual brines were prepared considering the hydration degree of each salt. NaCl was not included in the design but was maintained constant at 2.5% in the equilibrium.

Run	[NaCl] (g/L)	[KCl] (g/L)	[CaCl_2_] (g/L)	[MgCl_2_] (g/L)
1	25	5.0	10.0	10.0
2	25	15.0	10.0	0.0
3	25	15.0	0.0	10.0
4	25	5.0	10.0	10.0
5	25	10.0	5.0	10.0
6	25	15.0	5.0	5.0
7	25	8.3	8.3	8.3
8	25	15.0	0.0	10.0
9	25	15.0	10.0	0.0
10	25	13.3	3.3	8.3
11	25	13.3	8.3	3.3
12	25	11.7	6.7	6.7
13	25	10.0	5.0	10.0
14	25	10.0	10.0	5.0
Control	50	0.0	0.0	0.0

Notes: Concentrations expected at equilibrium. The Control run was packaged using the usual industrial conditions for salt, acidity, and pH.

**Table 2:** Study of the mineral bioaccessibility of green Spanish-style Manzanilla olives with K, Ca, and Mg chloride mixtures substituting 50% salt during packaging. Mineral concentrations in the raw material, supernatant, solid residue, and blank of the in vitro digestion, along with the respective average weights of replicates, necessary for the corresponding matter balances. Data are presented according to runs of the design.

Run	Fraction	[Na] mg/kg	[K] mg/kg	[Ca] mg/kg	[Mg] mg/kg	[P] mg/kg	Average Weight (g)
1	Raw material	6301.01 (21.64)	1571.64 (10.02)	4611.70 (3.80)	1570.25 (1.12)	59.25 (0.62)	2.0022 (0.0012)
	Supernatant	1176.90 (5.43)	108.02 (0.37)	62.86 (0.11)	86.18 (0.15)	16.64 (1.40)	33.2526 (0.3170)
	Solid residue	245.58 (9.32)	23.69 (0.95)	2876.60 (192.76)	151.89 (10.59)	26.88 (0.06)	2.6554 (0.3630)
	Blank	1041.36 (5.21)	21.57 (0.02)	1.94 (0.02)	4.62 (0.02)	23.47 (0.11)	26.3000 (N/A)
2	Raw material	6398.18 (32.66)	5003.36 (4.47)	4583.18 (4.49)	39.12 (1.15)	65.12 (0.52)	2.0212 (0.0004)
	Supernatant	1374.04 (3.58)	311.67 (1.17)	63.50 (0.17)	5.27 (0.01)	27.44 (0.52)	33.9886 (0.0093)
	Solid residue	413.22 (21.52)	107.45 (1.63)	3188.23 (64.31)	8.22 (0.06)	26.63 (0.06)	2.2075 (0.0042)
	Blank	1304.64 (1.80)	18.67 (0.11)	2.39 (0.01)	4.04 (0.01)	22.97 (0.06)	26.5800 (N/A)
3	Raw material	6503.01 (35.57)	5152.03 (11.76)	603.05 (3.21)	1556.03 (1.77)	62.17 (0.76)	2.0200 (0.0024)
	Supernatant	1200.95 (4.08)	325.18 (0.98)	10.24 (0.07)	78.10 (0.13)	19.05 (0.37)	32.8539 (0.2814)
	Solid residue	397.38 (2.69)	117.23 (2.31)	369.08 (6.79)	216.90 (6.32)	23.33 (0.03)	2.6643 (0.1004)
	Blank	1030.14 (1.18)	20.21 (0.13)	3.10 (0.05)	3.73 (0.01)	23.34 (0.11)	26.1800 (N/A)
4	Raw material	6512.51 (37.01)	1662.35 (6.28)	4664.07 (21.43)	1546.07 (19.54)	60.40 (0.35)	2.0093 (0.0050)
	Supernatant	1171.50 (3.45)	107.88 (0.14)	60.14 (0.67)	82.31 (0.22)	22.82 (0.60)	33.5133 (0.1219)
	Solid residue	304.18 (0.69)	40.02 (0.62)	3327.88 (111.28)	172.19 (2.42)	25.32 (0.12)	2.1750 (0.1122)
	Blank	1024.19 (0.85)	13.18 (0.09)	1.83 (0.01)	2.80 (0.01)	21.84 (0.03)	26.0900 (N/A)
5	Raw material	6561.75 (17.38)	3555.06 (21.64)	2744.65 (13.28)	1637.96 (5.26)	67.29 (0.21)	2.200 (0.0039)
	Supernatant	1205.01 (1.13)	227.20 (0.46)	45.86 (0.74)	88.14 (0.17)	27.69 (0.32)	33.6827 (0.0696)
	Solid residue	323.78 (9.94)	71.87 (2.04)	1899.09 (58.47)	169.77 (5.34)	26.61 (0.16)	2.0818 (0.0774)
	Blank	1057.39 (1.55)	20.38 (0.09)	1.95 (0.01)	4.05 (0.02)	23.18 (0.05)	26.3700 (N/A)
6	Raw material	6519.45 (21.43)	5066.09 (9.03)	2689.18 (26.97)	777.18 (2.04)	58.76 (0.34)	2.0106 (0.0004)
	Supernatant	1202.62 (1.70)	309.97 (1.07)	33.84 (0.14)	42.56 (0.08)	22.47 (0.47)	33.5629 (0.0913)
	Solid residue	296.74 (6.34)	107.36 (2.26)	1957.48 (34.97)	87.33 (3.60)	26.22 (0.05)	2.1235 (0.0664)
	Blank	1058.73 (3.16)	20.65 (0.09)	1.43 (0.01)	3.85 (0.03)	22.63 (0.01)	26.2800 (N/A)
7	Raw material	6451.19 (11.12)	2855.42 (6.25)	4064.94 (20.22)	1376.66 (5.95)	65.38 (0.68)	2.0080 (0.0017)
	Supernatant	1146.92 (1.29)	176.27 (1.13)	59.51 (0.36)	73.47 (0.25)	28.58 (1.07)	33.6337 (0.1532)
	Solid residue	293.33 (18.86)	71.77 (7.11)	3010.75 (116.49)	161.11 (10.98)	26.13 (0.01)	1.9928 (0.1410)
	Blank	996.4 (3.07)	18.21 (0.04)	2.06 (0.04)	3.45 (0.03)	22.43 (0.02)	26.0000 (N/A)
8	Raw material	6523.22 (10.66)	5058.85 (7.89)	577.51 (2.67)	1573.89 (5.00)	58.53 (0.63)	2.0219 (0.0014)
	Supernatant	1203.53 (2.85)	310.61 (0.81)	11.24 (0.03)	79.98 (0.61)	16.36 (0.10)	32.9357 (0.1282)
	Solid residue	263.17 (3.05)	91.07 (1.65)	313.04 (4.29)	195.00 (2.67)	26.60 (0.02)	2.8663 (0.0731)
	Blank	1028.28 (1.97)	21.51 (0.15)	2.75 (0.03)	3.56 (0.01)	23.47 (0.03)	26.3900 (N/A)
9	Raw material	6623.26 (13.64)	5085.61 (17.09)	4729.36 (20.19)	54.89 (0.34)	60.02 (1.17)	2.0240 (0.0116)
	Supernatant	1271.71 (2.05)	312.50 (3.15)	67.49 (1.60)	5.62 (0.01)	21.77 (0.61)	33.3218 (0.0891)
	Solid residue	365.01 (6.17)	111.28 (3.01)	2920.10 (64.53)	10.54 (0.25)	25.86 (0.02)	2.4397 (0.1109)
	Blank	1133.50 (1.64)	19.56 (0.21)	1.42 (0.04)	3.76 (0.01)	22.37 (0.02)	26.1800 (N/A)
10	Raw material	6627.00 (14.15)	4404.56 (14.10)	1973.31 (16.19)	1353.52 (6.19)	60.70 (0.05)	2.0226 (0.0068)
	Supernatant	1307.83 (1.58)	268.08 (0.27)	33.49 (0.75)	72.87 (0.09)	24.86 (0.80)	33.5741 (0.0680)
	Solid residue	306.94 (12.54)	81.97 (0.40)	1393.45 (70.29)	154.66 (2.87)	27.11 (0.14)	2.0773 (0.1266)
	Blank	1176.56 (0.92)	20.92 (0.04)	1.80 (0.13)	4.26 (0.01)	23.37 (0.02)	26.3000 (N/A)
11	Raw material	6603.18 (5.35)	4576.45 (30.24)	3963.80 (12.62)	591.53 (1.25)	64.11 (1.75)	2.0351 (0.0108)
	Supernatant	1322.01 (3.50)	286.46 (1.69)	58.48 (0.07)	35.21 (0.18)	24.78 (0.55)	33.9115 (0.0661)
	Solid residue	324.45 (4.62)	77.77 (6.02)	2734.00 (44.97)	59.19 (0.42)	25.87 (0.05)	2.1344 (0.0662)
	Blank	1199.41 (3.96)	17.77 (0.11)	1.32 (0.07)	3.83 (0.01)	22.50 (0.01)	26.5400 (N/A)
12	Raw material	6590.92 (19.17)	4040.13 (9.20)	3337.39 (5.87)	1107.05 (0.28)	63.02 (1.58)	2.0208 (0.0059)
	Supernatant	1198.58 (2.47)	253.07 (1.10)	39.46 (0.24)	62.21 (0.18)	22.21 (1.38)	33.7144 (0.0372)
	Solid residue	338.31 (13.78)	83.60 (1.98)	2373.68 (106.55)	114.34 (0.87)	26.41 (0.03)	2.2251 (0.2039)
	Blank	1066.51 (12.72)	18.81 (0.02)	1.06 (0.01)	3.59 (0.01)	22.63 (0.02)	26.0000 (N/A)
13	Raw material	6565.79 (12.91)	3355.81 (17.26)	2616.61 (4.02)	1577.68 (9.46)	65.10 (1.22)	2.0210 (0.0020)
	Supernatant	1265.22 (1.90)	215.17 (1.02)	38.12 (0.76)	88.68 (0.45)	25.77 (1.54)	33.7465 (0.2227)
	Solid residue	389.06 (8.62)	65.56 (1.14)	1858.10 (102.53)	134.39 (5.93)	26.30 (0.09)	2.2600 (0.2403)
	Blank	1144.76 (3.77)	21.33 (0.07)	2.77 (0.10)	3.74 (0.01)	22.69 (0.09)	26.2200 (N/A)
14	Raw material	6610.83 (15.63)	3381.24 (13.83)	4661.51 (15.06)	801.43 (3.06)	64.73 (0.96)	2.0159 (0.0078)
	Supernatant	1298.13 (8.16)	217.14 (0.59)	64.76 (0.33)	45.99 (0.21)	22.71 (1.10)	33.2502 (0.1086)
	Solid residue	244.24 (8.16)	54.51 (1.52)	3016.58 (84.48)	64.92 (2.72)	25.79 (0.06)	2.4151 (0.1632)
	Blank	1143.04 (1.46)	21.09 (0.06)	2.46 (0.03)	3.66 (0.03)	22.77 (0.06)	26.2800 (N/A)
Control	Raw material	13590.21 (97.57)	99.49 (1.53)	608.58 (5.07)	49.46 (0.10)	68.35 (0.89)	2.0080 (0.0037)
	Supernatant	1626.03 (2.73)	21.56 (0.01)	10.79 (0.13)	5.20 (0.01)	25.04 (1.00)	33.6133 (0.0306)
	Solid residue	453.52 (12.34)	4.50 (0.13)	411.69 (15.88)	9.60 (0.16)	27.15 (0.02)	2.2144 (0.1392)
	Blank	1076.39 (5.52)	20.31 (0.10)	1.49 (0.01)	3.61 (0.01)	23.47 (0.05)	25.9800 (N/A)

Notes: Values are the average of three replicates. Standard error in parenthesis; Supernatant corresponds to that from the standard protocol + the additional re-extractions; N/A, not applicable.

**Table 3:** Study of the mineral bioaccessibility of plain green Spanish-style Manzanilla olives with K, Ca, and Mg chloride mixtures substituting 50% salt during packaging. Na, K, Ca, Mg, and P percentages in the supernatant solution (S), solid residue (SR), and total recovery (TR) after digestion of the olive pulp, according to the experimental runs.

Run	Na (S)	Na (SR)	Na (TR)	K (S)	K (SR)	K (TR)	Ca (S)	Ca (SR)	Ca (TR)	Mg (S)	Mg (SR)	Mg (TR)	P (S)	P (SR)	P (TR)
1	93.06 (0.49)	5.09 (0.25)	98.16 (0.42)	96.12 (0.77)	1.96 (0.05)	98.08 (0.73)	22.08 (0.08)	79.70 (0.52)	101.79 (0.55)	87.29 (0.57)	12.34 (0.08)	99.63 (0.64)	61.78 (0.60)	35.65 (0.81)	97.43 (0.62)
2	92.97 (0.59)	7.01 (0.27)	99.98 (0.67)	99.83 (0.36)	2.34 (0.02)	102.17 (0.37)	22.61 (0.03)	75.80 (0.39)	98.41 (0.42)	78.38 (0.65)	22.93 (0.29)	101.32 (0.68)	55.44 (0.33)	45.93 (0.48)	101.36 (0.39)
3	95.03 (0.41)	8.05 (0.12)	103.08 (0.35)	97.58 (0.70)	3.01 (0.11)	100.59 (0.60)	20.38 (0.23)	80.51 (0.38)	100.89 (0.37)	78.53 (0.48)	18.42 (0.78)	96.96 (0.35)	61.46 (0.77)	40.36 (0.73)	101.82 (0.71)
4	95.83 (1.06)	5.06 (0.13)	100.88 (0.98)	97.95 (0.29)	2.61 (0.09)	100.56 (0.22)	21.00 (0.29)	76.71 (0.56)	97.71 (0.66)	86.46 (0.44)	12.03 (0.25)	98.49 (0.19)	58.73 (0.86)	40.72 (0.63)	99.45 (0.59)
5	95.85 (0.35)	5.06 (0.08)	100.91 (0.41)	99.08 (0.17)	2.08 (0.03)	101.16 (0.19)	26.93 (0.47)	70.99 (0.82)	97.92 (0.43)	86.50 (0.22)	10.64 (0.17)	97.14 (0.25)	57.66 (1.25)	42.44 (1.11)	100.09 (0.67)
6	95.66 (0.19)	4.80 (0.09)	100.46 (0.21)	96.81 (0.32)	2.23 (0.02)	99.04 (0.33)	20.31 (0.11)	76.71 (0.26)	97.02 (0.20)	84.94 (0.20)	11.81 (0.32)	96.75 (0.17)	59.44 (0.62)	40.31 (0.53)	99.76 (0.86)
7	97.87 (0.58)	4.43 (0.12)	102.30 (0.65)	95.13 (0.45)	2.43 (0.15)	97.56 (0.42)	23.86 (0.16)	72.72 (0.46)	96.58 (0.32)	86.15 (0.39)	11.41 (0.40)	97.56 (0.35)	57.29 (1.02)	42.93 (0.36)	100.22 (1.24)
8	94.79 (0.63)	5.72 (0.07)	100.51 (0.57)	94.47 (0.23)	2.55 (0.05)	97.02 (0.22)	25.50 (0.13)	76.76 (0.56)	102.25 (0.64)	79.82 (0.56)	17.54 (0.10)	97.36 (0.58)	59.78 (1.36)	39.62 (0.46)	99.40 (0.94)
9	94.73 (0.37)	6.62 (0.04)	101.36 (0.40)	96.17 (0.76)	2.63 (0.05)	98.80 (0.73)	23.09 (0.50)	74.14 (0.50)	97.23 (0.39)	80.01 (0.20)	23.05 (0.18)	103.06 (0.34)	56.30 (1.30)	43.51 (0.33)	99.81 (1.14)
10	96.84 (0.38)	4.79 (0.32)	101.62 (0.25)	94.85 (0.22)	1.91 (0.07)	96.76 (0.16)	26.97 (0.58)	71.67 (1.49)	98.65 (0.93)	85.28 (0.27)	11.69 (0.21)	96.97 (0.20)	58.24 (0.53)	41.73 (0.23)	99.97 (0.56)
11	96.73 (0.67)	5.15 (0.03)	101.87 (0.66)	99.24 (0.63)	1.77 (0.13)	101.02 (0.57)	24.15 (0.04)	72.20 (0.19)	96.35 (0.18)	90.74 (0.68)	10.49 (0.12)	101.22 (0.77)	58.66 (0.70)	40.44 (0.35)	99.10 (0.65)
12	95.68 (0.48)	5.74 (0.43)	101.41 (0.39)	99.01 (0.43)	2.30 (0.14)	101.31 (0.56)	19.41 (0.10)	77.42 (0.26)	96.83 (0.28)	90.02 (0.18)	11.29 (0.14)	101.31 (0.13)	60.13 (0.96)	38.17 (0.71)	98.30 (0.62)
13	95.56 (0.77)	6.57 (0.22)	102.13 (0.56)	98.81 (0.38)	2.17 (0.08)	100.98 (0.36)	22.94 (0.40)	77.57 (0.81)	100.50 (0.52)	90.77 (0.28)	9.35 (0.05)	100.11 (0.31)	57.96 (0.68)	43.17 (0.71)	101.13 (0.30)
14	98.47 (0.22)	4.40 (0.12)	102.87 (0.28)	97.80 (0.55)	1.92 (0.07)	99.73 (0.57)	22.23 (0.16)	76.96 (0.85)	99.19 (0.71)	88.70 (0.35)	9.60 (0.11)	98.29 (0.39)	60.91 (0.58)	41.49 (0.63)	102.40 (0.66)
Control	97.81 (0.44)	3.70 (0.20)	101.51 (0.28)	98.66 (0.24)	5.02 (0.30)	103.68 (0.26)	26.52 (0.39)	73.89 (0.56)	100.40 (0.77)	81.48 (0.29)	21.37 (0.67)	102.85 (0.49)	60.77 (1.21)	40.01 (0.50)	100.79 (1.11)

Notes: Data are the average of three replicates, standard error in parenthesis.

**Table 4:** K, Ca, and Mg amounts in the pulp; bioaccessible amounts; and their contributions to the RDI. Differences (and percentages) between labelled mineral contents in the pulp and the bioaccessible contents. Amounts are expressed as mg/100 g pulp. The information is presented according to experimental runs. Anal., analytical; Bioac., bioaccessible; Label., labelling.

Design Run	Comparison between Analytical and Bioaccessible K Content				Comparison between Analytical and Bioaccessible Ca Content						Comparison between Analytical and Bioaccessible Mg Content					
	Anal. Content		Bioac. Content		Anal. Content		Bioac. Content		Label. Differences		Anal. Content		Bioac. Content		Label. Differences	
	Amount	RDI (%)	Amount	RDI (%)	Amount	RDI (%)	Amount	RDI (%)	Amount	Percentage	Amount	RDI (%)	Amount	RDI (%)	Amount	Percentage
1	157.164 (1.002)	7.85 (0.07)	151.1 (1.2)	7.55 (0.06)	461.170 (0.380)	57.64 (0.03)	101.8 (0.4)	12.73 (0.04)	359.370	77.93	157.025 (0.112)	41.87 (0.01)	137.1 (0.9)	36.55 (0.24)	19.93	12.69
2	500.336 (0.447)	25.02 (0.03)	499.5 (1.8)	24.97 (0.09)	458.318 (0.449)	57.89 (0.03)	103.6 (0.2)	12.95 (0.02)	354.718	77.40	3.912 (0.115)	1.04 (0.01)	3.1 (<0.1)	0.82 (<0.01)	0.81	20.76
3	515.203 (1.176)	25.76 (0.08)	502.7 (3.6)	25.14 (0.18)	60.305 (0.321)	7.54 (0.02)	12.3 (0.1)	1.54 (0.02)	48.005	79.60	155.603 (0.177)	41.49 (0.02)	122.2 (0.7)	32.59 (0.20)	33.43	21.48
4	166.235 (0.628)	8.312 (0.04)	162.8 (0.5)	8.14 (0.02)	466.407 (2.143)	58.30 (0.14)	98.0 (1.3)	12.25 (0.17)	368.407	78.99	154.607 (1.954)	41.23 (0.13)	133.7 (0.7)	35.64 (0.18)	20.91	13.52
5	355.506 (2.164)	17.78 (0.14)	352.2 (0.6)	17.61 (0.03)	274.465 (1.328)	34.31 (0.09)	73.9 (1.3)	9.24 (0.16)	200.565	73.07	163.796 (0.526)	43.68 (0.04)	141.7 (0.4)	37.78 (0.10)	22.07	13.47
6	506.609 (0.903)	25.33 (0.06)	490.4 (1.6)	24.52 (0.08)	268.918 (2.697)	33.61 (0.18)	54.6 (0.3)	6.83 (0.04)	214.318	79.70	77.718 (0.204)	20.72 (0.01)	66.0 (0.2)	17.60 (0.04)	11.72	15.08
7	285.542 (0.625)	14.28 (0.04)	271.6 (1.3)	13.58 (0.06)	406.494 (2.022)	50.81 (0.13)	97.0 (0.7)	12.13 (0.08)	309.494	76.14	137.666 (0.595)	36.71 (0.04)	118.6 (0.5)	31.62 (0.14)	19.07	13.85
8	505.885 (0.789)	25.29 (0.05)	477.9 (1.1)	23.89 (0.06)	57.751 (0.267)	7.22 (0.02)	14.7 (0.1)	1.84 (0.01)	43.051	74.55	157.389 (0.500)	41.97 (0.03)	125.6 (0.9)	33.50 (0.23)	31.79	20.20
9	508.561 (1.709)	25.43 (0.11)	489.1 (3.8)	24.45 (0.19)	472.936 (2.019)	59.12 (0.13)	109.2 (2.4)	13.65 (0.30)	363.736	76.91	5.489 (0.034)	1.46 (<0.01)	4.4 (<0.1)	1.17 (<0.01)	1.09	19.84
10	440.456 (1.410)	22.02 (0.09)	417.8 (1.0)	20.89 (0.05)	197.331 (1.619)	24.67 (0.11)	53.2 (1.1)	6.65 (0.14)	144.131	73.04	135.352 (0.619)	36.09 (0.04)	115.4 (0.4)	30.78 (0.10)	19.95	14.74
11	457.645 (3.024)	22.88 (0.20)	454.2 (2.9)	22.71 (0.14)	396.380 (1.262)	49.55 (0.08)	95.7 (0.2)	11.97 (0.02)	300.680	75.86	59.153 (0.125)	15.77 (<0.01)	53.7 (0.4)	14.31 (0.11)	5.45	9.22
12	404.013 (0.920)	20.20 (0.06)	400.0 (1.8)	20.00 (0.09)	333.739 (0.587)	41.72 (0.04)	64.8 (0.3)	8.10 (0.04)	268.939	80.58	110.705 (0.028)	29.52 (<0.01)	99.7 (0.2)	26.58 (0.05)	11.01	9.94
13	335.581 (1.726)	16.78 (0.12)	331.6 (1.3)	16.60 (0.06)	261.661 (0.402)	32.71 (0.03)	60.0 (1.0)	7.50 (0.13)	201.661	77.37	157.768 (0.946)	42.07 (0.06)	143.2 (0.4)	38.19 (0.12)	14.57	9.23
14	338.124 (1.383)	16.91 (0.09)	330.7 (1.9)	16.53 (0.09)	466.151 (1.506)	58.27 (0.10)	103.6 (0.7)	12.95 (0.10)	362.551	77.78	80.143 (0.306)	21.37 (0.02)	71.1 (0.3)	18.96 (0.07)	9.05	11.29
Control	9.949 (0.153)	0.50 (0.01)	9.8 (<0.1)	0.49 (<0.01)	60.858 (0.507)	7.61 (0.03)	16.1 (0.2)	2.02 (0.03)	44.758	73.54	4.946 (0.010)	1.32 (<0.01)	4.0 (<0.1)	1.07 (<0.01)	0.95	19.13

Notes: Data are averages of three replicates; standard error in parenthesis. Reference daily intake (RDI) is based on the following values (mg/day): K, 2000; Ca, 800; Mg, 375; (Regulation (EU)169/2011).

**Table 5:** Na and P amounts in the pulp, bioaccessible amounts, and their contributions to the RDI. Amounts are expressed as mg/100 g pulp. The information is presented according to experimental runs.

Design Run	Comparison between Analytical and Bioaccessible Na Content				Comparison between Analytical and Bioaccessible P Content			
	Analytical Content		Bioaccessible Content		Analytical Content		Bioaccessible Content	
	Amount	RDI (%)	Amount	RDI (%)	Amount	RDI (%)	Amount	RDI (%)
1	630.10 (2.16)	26.25 (0.66)	586.4 (3.1)	25.50 (0.13)	5.925 (0.062)	0.85 (<0.01)	3.67 (0.04)	0.52 (<0.01)
2	639.82 (3.27	26.66 (0.13)	594.8 (3.8)	25.86 (0.16)	6.512 (0.052)	0.93 (<0.01)	3.61 (0.02)	0.52 (<0.01)
3	650.301 (3.557)	27.10 (0.20)	618.0 (2.7)	26.87 (0.12)	6.217 (0.076)	0.89 (<0.01)	3.82 (0.05)	0.55 (0.01)
4	651.251 (3.701)	27.14 (0.27)	624.1 (6.9)	27.13 (0.30)	6.040 (0.035)	0.86 (<0.01)	3.55 (0.05)	0.51 (0.01)
5	656.175 (1.738)	27.34(0.33)	628.9 (2.3)	27.35 (0.10)	6.729 (0.021)	0.96 (<0.01)	3.88 (0.08)	0.55 (0.01)
6	651.945 (2.143)	27.16 (0.40)	623.7 (1.2)	27.12 (0.05)	5.876 (0.034)	0.84 (<0.01)	3.49 (0.04)	0.50 (0.01)
7	645.119 (1.112)	26.89 (0.47)	631.4 (3.7)	27.45 (0.16)	6.538 (0.068)	0.93 (<0.01)	3.75 (0.07)	0.54 (0.01)
8	652.322 (1.066)	27.18 (0.53)	618.4 (4.1)	26.88 (0.18)	5.853 (0.063)	0.84 (<0.01)	3.50 (0.08)	0.50 (0.01)
9	662.326 (1.364)	27.60 (0.60)	627.4 (2.4)	27.28 (0.11)	6.002 (0.117)	0.86 (<0.01)	3.38 (0.08)	0.48 (0.01)
10	662.700 (1.415)	27.61 (0.68)	641.7 (2.5)	27.90 (0.11)	6.070 (0.005)	0.87 (<0.01)	3.54 (0.04)	0.51 (<0.01)
11	660.318 (0.535)	27.51 (0.73)	638.7 (4.4)	27.77 (0.19)	6.411 (0.175)	0.92 (0.01)	3.76 (0.04)	0.54 (0.01)
12	659.092 (1.917)	27.46 (0.80)	630.6 (3.2)	27.42 (0.14)	6.302 (0.158)	0.90 (0.01)	3.79 (0.06)	0.54 (0.01)
13	656.579 (1.291)	27.36 (0.87)	627.4 (5.0)	27.28 (0.22)	6.510 (0.122)	0.93 (0.01)	3.77 (0.04)	0.54 (0.01)
14	661.083 (1.563)	27.55 (0.93)	651.0 (1.4)	28.30 (0.06)	6.473 (0.096)	0.92 (0.01)	3.94 (0.04)	0.56 (0.01)
Control	1359.021 (9.757)	56.63 (0.98)	1329.3 (6.0)	55.38 (0.26)	6.835 (0.089)	0.98 (0.01)	4.16 (0.08)	0.60 (0.01)

Notes: Data are the averages of three replicates; standard error in parenthesis. Reference daily intake (RDI) is based on the following values (mg/day): Na, 2400; P, 700; (Regulation (EU)169/2011).

## Data Availability

The original contributions presented in the study are included in the article/Supplementary Materials, further inquiries can be directed to the corresponding author.

## Supplementary Materials

The following supporting information can be downloaded at: https://www.mdpi.com/article/10.3390/foods13172671/s1, Table S1: Effect of fortification with KCl, CaCl_2_, and MgCl_2_ on the bioaccessibility of the main mineral nutrients in plain green Spanish-style Manzanilla table olives. Comparison of the results from applying the standard Crews and Miller protocols and those incorporating one or two additional washes. The table shows the concentrations in the raw material, supernatant after treatment, solid residue, and blank, as well as the corresponding percentages of bioaccessibility, proportion in the final solid residue, and the total recovery based on the matter balance; Table S2: Effect of fortification with KCl, CaCl_2_, and MgCl_2_ on the bioaccesibility of the main mineral nutrients in plain green Spanish-style Manzanilla table olives. Comparison of the results between the standard Crews and Miller protocols and those incorporating one or two additional washes. Average weight of the raw material, supernatant, solid residue after digestion, and blank, considered for estimating mineral bioaccessibilities and matter balance.
