# Supplementary material for: Bioaccessibility of Mineral Nutrients in Plain Green Spanish-Style Manzanilla Table Olives Packaged in Nutrient Salt Mixtures
Source: Foods. 2024 Aug 24;13(17):2671. doi: 10.3390/foods13172671 (PMC11394259; doi:10.3390/foods13172671)
Supplement: Supplementary file 1 [file foods-13-02671-s001.zip › foods-3160471-supplementary.pdf]

Supplementary material to

Bioaccessibility of Mineral Nutrients in Plain Green Spanish-Style Manzanilla Table Olives Packaged in Nutrient Salt Mixtures

Table S1. Effect of fortification in KCl, $CaCl_2$ and $MgCl_2$ on the bioaccessibility of the main mineral nutrients in plain green Spanish-style Manzanilla table olives. Comparison of the results from applying the standard Crews and Miller protocols and those incorporating one or two additional washes. The table shows the concentrations in the raw material, supernatant after the treatment, solid residue, and blank, as well as the corresponding percentages of bioaccessibility, proportion in the final solid residue, and the total recovery based on the matter balance.

Protocol	Treatment	Mineral nutrient	Raw material	Supernatant	Solid residue	Blank	Bioaccessibility (%)	Solid residue (%)	Total recovery (%)
Crews	No washes	Na	26238 (334)	10830 (80)	10311 (311)	6350 (31)	64.08 (1.77)	38.78 (1.72)	102.85 (0.10)
Crews	1 wash	Na	26238 (334)	6217 (36)	3859 (40)	6350 (31)	86.32 (1.52)	15.09 (1.64)	101.41 (1.79)
Crews	2 washes	Na	26238 (334)	3876 (5)	863 (20)	5472 (52)	93.72 (0.18)	3.39 (0.23)	97.11 (0.17)
Crews	No washes	K	540 (8)	108 (1)	67 (4)	13 (1)	89.72 (0.19)	12.37 (1.60)	102.09 (1.69)
Crews	1 wash	K	540 (8)	59 (1)	41 (2)	13 (1)	94.46 (0.48)	7.61 (0.14)	102.07 (0.62)
Crews	2 washes	K	540 (8)	45 (1)	18 (1)	16 (1)	98.93 (0.39)	3.47 (0.26)	102.40 (0.50)
Crews	No washes	Ca	818 (2)	1.2 (0.1)	780 (40)	0.6 (0.1)	0.33 (0.07)	95.56 (0.35)	95.89 (0.36)
Crews	1 wash	Ca	818 (2)	1.2 (0.1)	782 (52)	0.6 (0.1)	1.06 (0.14)	95.27 (0.49)	96.33 (0.38)
Crews	2 washes	Ca	818 (2)	0.5 (0.1)	711 (2)	0.5 (0.1)	8.98 (0.42)	89.39 (1.25)	98.37 (0.89)
Crews	No washes	Mg	119.3 (1.2)	14.4 (0.1)	65 (2)	2.4 (0.1)	51.22 (1.56)	53.27 (1.69)	104.50 (0.16)
Crews	1 wash	Mg	119.3 (1.2)	8.7 (0.1)	51 (3)	2.4 (0.1)	61.42 (1.94)	42.79 (1.87)	104.21 (0.24)
Crews	2 washes	Mg	119.3 (1.2)	8.4 (0.4)	39.3 (0.7)	5.3 (0.01)	67.99 (1.02)	33.83 (0.65)	101.82 (0.92)
Crews	No washes	P	107.5 (0.9)	58.4 (0.5)	45.6 (1.3)	38.5 (0.1)	59.20 (1.36)	41.91 (2.28)	101.11 (0.97)
Crews	1 wash	P	107.5 (0.9)	31.5 (0.5)	30.9 (1.8)	38.5 (0.1)	66.01 (0.92)	28.72 (0.38)	94.73 (1.16)
Crews	2 washes	P	107.5 (0.9)	21.8 (0.1)	41.5 (0.1)	41.5 (0.1)	68.03 (0.34)	33.81 (0.40)	101.83 (0.36)
Miller	No washes	Na	26238 (334)	3859 (5)	3563 (45)	1934 (12)	81.69 (0.55)	14.47 (0.70)	96.16 (0.15)
Miller	1 wash	Na	26238 (334)	2896 (11)	672 (30)	1934 (12)	92.77 (0.17)	2.39 (0.30)	95.16 (0.20)
Miller	2 washes	Na	26238 (334)	2391 (8)	158 (4)	2006 (15)	95.40 (0.16)	0.61 (0.08)	96.02 (0.18)
Miller	No washes	K	540 (8)	59.7 (0.1)	58.4 (0.8)	19.8 (0.1)	86.71 (0.44)	11.48 (0.04)	98.19 (0.44)
Miller	1 wash	K	540 (8)	44.1 (0.1)	25.6 (0.5)	19.8 (0.1)	92.71 (1.05)	4.40 (0.30)	97.11 (0.79)
Miller	2 washes	K	540 (8)	35.3 (0.1)	2.4 (0.1)	15.1 (0.1)	98.05 (0.07)	0.44 (0.02)	98.49 (0.06)
Miller	No washes	Ca	818 (2)	10.3 (0.1)	657 (0.5)	1.4 (0.1)	13.25 (0.31)	85.32 (0.99)	98.58 (0.79)
Miller	1 wash	Ca	818 (2)	9.4 (0.1)	722.3 (9.5)	1.4 (0.1)	17.66 (0.62)	81.65 (1.40)	99.31 (1.38)
Miller	2 washes	Ca	818 (2)	7.1 (0.1)	653.7 (33.3)	1.8 (0.1)	17.00 (0.21)	79.95 (1.36)	96.95 (1.16)
Miller	No washes	Mg	119.3 (1.2)	11.3 (0.1)	28.9 (0.5)	3.8 (0.1)	73.95 (0.20)	25.77 (0.85)	99.72 (0.70)
Miller	1 wash	Mg	119.3 (1.2)	9.1 (0.1)	16.7 (0.2)	3.8 (0.1)	89.60 (0.77)	13.01 (0.66)	102.61 (0.88)
Miller	2 washes	Mg	119.3 (1.2)	8.1 (0.1)	9.6 (0.1)	4.1 (0.1)	90.98 (1.23)	8.19 (0.76)	99.18 (0.47)
Miller	No washes	P	107.5 (0.9)	31.0 (0.1)	37.1 (0.4)	23.5 (0.1)	59.73 (0.60)	36.66 (0.58)	96.40 (0.16)
Miller	1 wash	P	107.5 (0.9)	22.3 (0.1)	35.1 (0.5)	23.5 (0.1)	65.71 (0.44)	30.24 (1.08)	95.95 (1.08)
Miller	2 washes	P	107.5 (0.9)	17.1 (0.1)	37.8 (2)	21.9 (0.1)	67.24 (0.70)	35.11 (0.24)	102.35 (0.58)

Notes: Values are average of three independent replicate; standard error in parenthesis. As the olives used for the two protocols were from the same bath, their minerals concentrations were the same.

Table S2. Effect of fortification with KCl, $CaCl_2$ and $MgCl_2$ on the bioaccessibility of the main mineral nutrients in plain green Spanish-style Manzanilla table olives. Comparison of the results between the standard Crews and Miller protocols and those incorporating one or two additional washes. Average weight of the raw material, supernatant, solid residue after digestion, and blank considered for estimating mineral bioaccessibilities and matter balance.

Protocol	Treatment	Raw material weight (g)	Supernatant* (g)	Solid residue weight (g)	Blank weight (g)
Crews	No washes	25.03 (0.02)	133.08 (2.9)	25.03 (2.75)	160.54
Crews	1 wash	25.05 (0.01)	255.3 (2.1)	25.78 (3.08)	160.54
Crews	2 washes	25.05 (0.01)	361.7 (0.7)	25.78 (0.50)	147.20
Miller	No washes	2.0012 (0.0003)	24.75 (0.05)	2.1299 (0.0534)	27.20
Miller	1 wash	2.0005 (0.0002)	34.988 (0.048)	1.8542 (0.0654)	27.20
Miller	2 washes	2.0032 (0.0009)	44.3191 (0.3095)	2.0502 (0.2192)	27.85

Notes: Values are the average of three replicates. Standard error in parenthesis; *supernatant + washing water if applied.
